# The neurobehavioural effects of cannabidiol in alcohol use disorder: Study protocol for a double-blind, randomised, cross over, placebo-controlled trial

**DOI:** 10.1016/j.conctc.2024.101341

**Published:** 2024-08-13

**Authors:** Tristan P. Hurzeler, Warren Logge, Joshua Watt, Marilena M. DeMayo, Anastasia Suraev, Iain S. McGregor, Paul S. Haber, Kirsten C. Morley

**Affiliations:** a University of Sydney, Faculty of Medicine and Health, Sydney Medical School, NSW, Australia; b Edith Collins Centre for Translational Research, Royal Prince Alfred Hospital, NSW, Australia; c Department of Radiology and Department of Psychiatry, University of Calgary, Calgary, AB, Canada; d University of Sydney, Lambert Initiative for Cannabinoid Therapeutics, Sydney, NSW, Australia; e University of Sydney, Faculty of Science, School of Psychology, Sydney, NSW, Australia

**Keywords:** Cannabidiol, Alcohol use disorder, Mechanisms, Alcohol dependence, Neuroimaging, Psychophysiology, fMRI

## Abstract

Current treatments for alcohol use disorders (AUD) have limited efficacy. Recently, Cannabidiol (CBD) has been examined in a multitude of clinical settings. Preclinical and clinical results suggest that CBD might be particularly well suited for the treatment of AUD and may reduce alcohol cue and stress-induced craving and alcohol seeking. This study aims to investigate this new pharmacotherapy with a particular focus on neurobiological and physiological indicators of craving. *Methods*: In this double-blind, within-subject, randomised, placebo-controlled, cross-over study, non-treatment seekers will be randomly allocated to three days of four 200 mg CBD gel capsules (800 mg/day) or placebo, with an 18-day washout period. Cognitive, clinical, and neuroimaging assessments will be completed during these three days. The CBD and placebo assessments will be compared. The primary outcomes are i) BOLD signal as a proxy for regional activity during a cue reactivity and a fear response task measured with functional magnetic resonance imaging (fMRI), ii) heart rate variability and skin conductance levels as a proxy for psychophysiological responses to alcohol stimuli. The secondary outcomes are: i) neurometabolite levels (γ-Aminobutyric acid, ethanol, glutathione, and glutamate + glutamine (combined signal)) using magnetic resonance spectroscopy (MRS); ii) functional connectivity using resting state fMRI (rsfMRI); iii) executive functioning task results; iv) clinical outcomes such as craving, anxiety, and sleep. *Discussion:* This study will improve the understanding of the mechanisms of action of CBD and provide early signals of efficacy regarding the therapeutic potential of CBD in the treatment of alcohol use disorder.

ClinicalTrials.gov Identifier: NCT05387148.

## 1 Introduction

Alcohol use disorder (AUD) is a chronic and relapsing disorder that is a major public health concern due to the associated medical, psychological, and social sequelae. Although pharmacological is now a widely accepted approach to the management of AUD, current treatments have modest efficacy whereby new treatment approaches are required [1]. Cannabidiol (CBD), a cannabis compound that lacks any intoxicating effects, has been suggested as a potential pharmacotherapeutic in a multitude of clinical contexts [2,3] and may have therapeutic potential in managing AUD. CBD inhibits fatty acid-binding proteins (FABP) catabolism of anandamide (AEA) and reduces cellular uptake of endocannabinoids [4]. Previous research suggests that the endocannabinoid system may underlie psychiatric and substance use disorders [5]. Indeed, the endocannabinoid system has been found to be perturbed following chronic heavy alcohol use [6] and is implicated in reward processing [7].

**Table 1:**
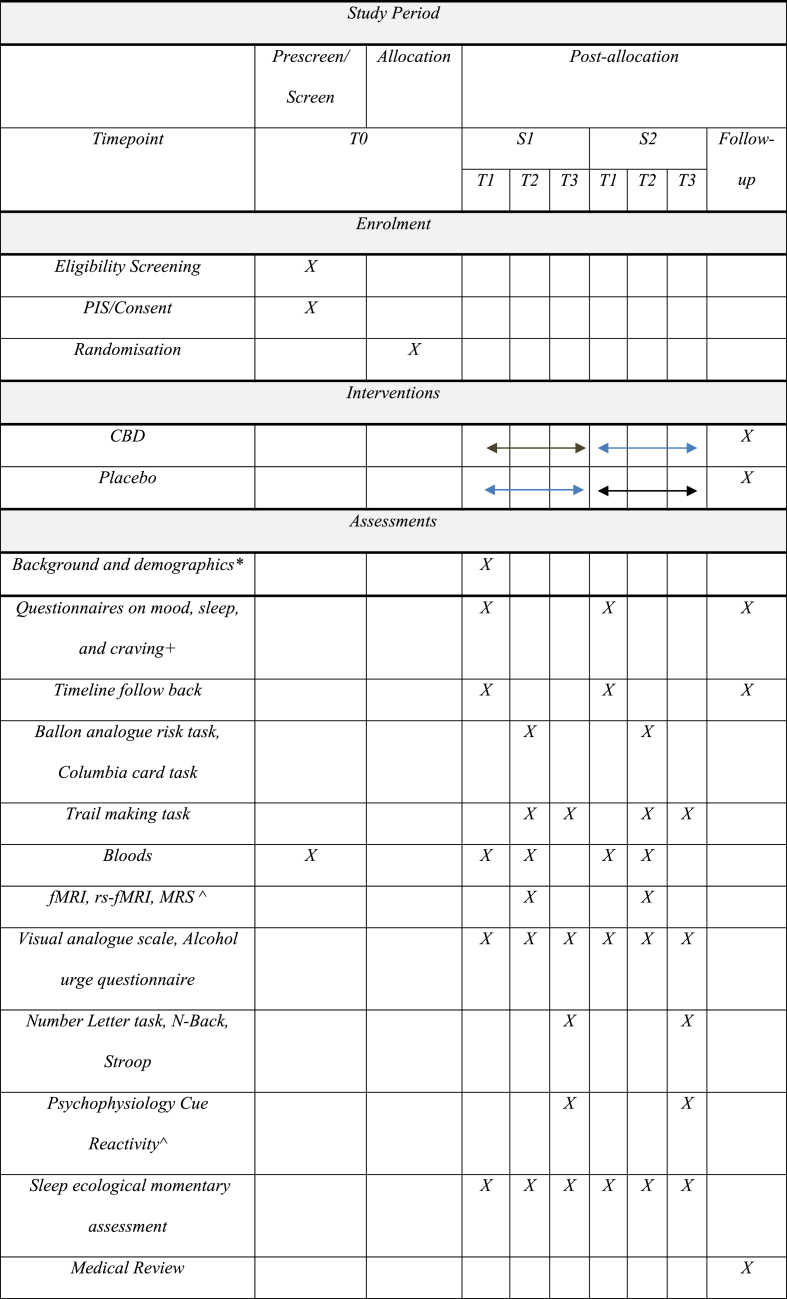
Spirit schedule.

* Demographics, medical history, personal and family history of AUD, and alcohol treatment history.

+Timeline follow-back method (TLFB), Alcohol Dependence Scale (ADS), Penn Alcohol Craving Scale (PACS), the Drinker Inventory of Consequences (DrInC-L), Tension Reduction Alcohol Outcome Expectancies (TRAE), Depression Anxiety symptom scale DASS, Insomnia Severity Index (ISI), Alcohol Abstinence Self-Efficacy (AASE), consequences of drinking (DrInC); Obsessive Compulsive Drinking Scale (OCDS), Behavioural approach and avoidance (BIS/BAS), Intolerance of Uncertainty Scale (IUS), Impulsivity Behaviour Scale (UPPS), expectancy of alcohol effects and urges to drink (AUQ), Positive and Negative Affect Schedule (PANAS), Visual Analogue Scales (VAS) assessing alcohol craving, thirst, and anxiety.

^See details in measures section.

# *Leeds Sleep Evaluation Questionnaire (LSEQ), Consensus Sleep Diary (CSD-M) through the SEMA3 application*.).

CBD modulates the endocannabinoid system through negative allosteric modulation of the cannabinoid 1 receptor (CB1R) [8]. Preclinical research has demonstrated that CBD not only modulates the endocannabinoid system but also serotoninergic, dopaminergic, glutamatergic, and γ-aminobutyric acidergic (GABA) signalling [9,10]. This poly-pharmaceutical action of CBD may explain the various therapeutic properties including anti-seizure [11], anxiolytic [[12], [13], [14], [15], [16], [17]], neuroprotective [18], anti-inflammatory and antioxidant effects of CBD [5,19,20]. Preclinical research has demonstrated that CBD administration reduces stress and drug cue alcohol reinstatement, voluntary alcohol consumption, withdrawal symptoms, and alcohol-induced relapse behaviours [21,22]. In clinical samples, CBD has been shown to reduce nicotine consumption in tobacco smokers [23] and cue-induced craving and anxiety in opioid dependent individuals [24]. These results suggesting that CBD may modulate cue-elicited motivational urges and drug-seeking behaviours [25,26].

Cue-induced craving is a substantial contributor to relapse [27]. fMRI task-based studies that elicit craving implicate specific regions leading them to be considered to be associated with cue and stress-induced craving. These regions include the posterior insula, posterior and anterior cingulate, medial prefrontal areas, and the striatum which, when more active in cue-inducing imaging tasks, have also been associated with increased rates of relapse [28]. Various studies have demonstrated that CBD modulates activity in these regions in samples of healthy participants [29]. While several trials investigating the potential therapeutic properties of CBD for AUD have recently been completed or are ongoing (NCT04873453 and NCT03252756), the effects of CBD on regional brain activity in those with AUD has yet to be comprehensively elucidated. Additionally, while cue induced-craving and drug cue-induced changes to heart rate and salivary cortisol have been shown to be modulated by 400 mg CBD in a sample of heroin users [24] these experimental paradigms have not been explored in AUD samples.

We aimed to conduct a double-blind, within-subject cross-over, randomised trial in individuals with AUD to determine the effect of CBD versus placebo on i) blood-oxygen-level-dependent functional magenetic resonance imaging (BOLD fMRI) and ii) psychophysiological (heart rate variability and skin conductance) responses to alcohol and threat stimuli. Secondary objectives include examination of the effect of CBD versus placebo on i) neurometabolite levels using magnetic resonance spectroscopy; ii) functional connectivity using resting state fMRI (rsfMRI); iii) self-reported alcohol craving, mood, and sleep; and iv) cognitive functioning.

## 2 Methods

### 2.1 Design

**Fig. 1:**
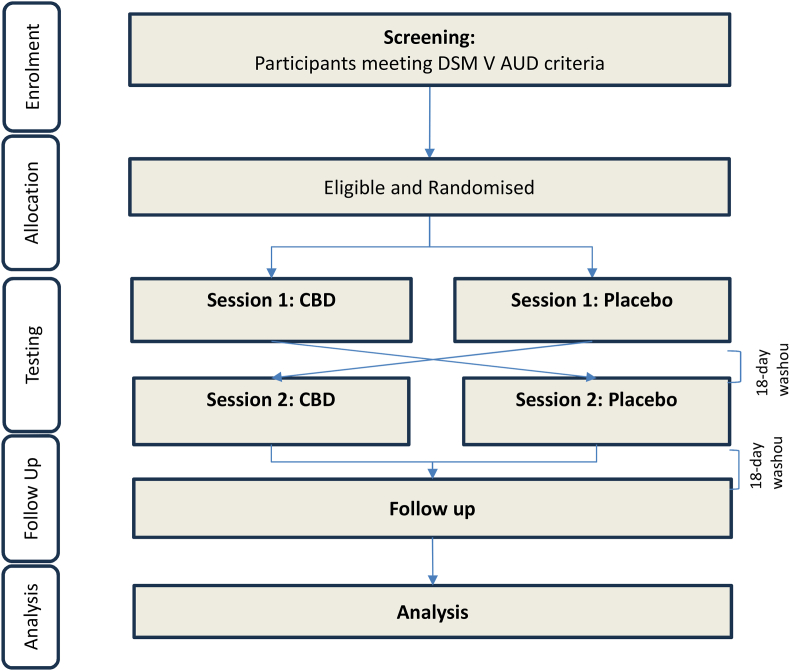
CONSORT flow diagram.

This single-center, within-subject, cross-over, double-blind, randomised trial with 3 days of 800 mg CBD/matched placebo will be conducted at the Royal Prince Alfred Hospital (RPAH) in Sydney, NSW, Australia. 22 non-treatment-seeking individuals who meet DSM-V criteria for current alcohol use disorder, but are not engaged in any form of AUD treatment currently (<60 days) nor seeking treatment for AUD at the time of recruitment, will be recruited to participate in the research. Participants will be recruited through clinical referral from treating physicians, nurses, and psychologists among RPAH outpatients as well as via flyers/community advertisements and through websites. On the second day of each arm, participants will be scanned 1.5 h after treatment administration, corresponding with C_max_ occurring between 0 and 4 h after CBD administration. Further, a conservative washout period of 18 days was selected between each testing session and follow-up as the half-life of CBD following chronic administration occurs between 2 and 5 days [30]. More recent literature suggests that on average a 13-day washout period would reduce CBD plasma levels to ‘zero’ at a 300 mg dose [31]. Fig. 1 indicates the flow of participants throughout the study while Table 1 describes the spirit schedule of specific measures through the duration of the study.

**Fig. 2:**
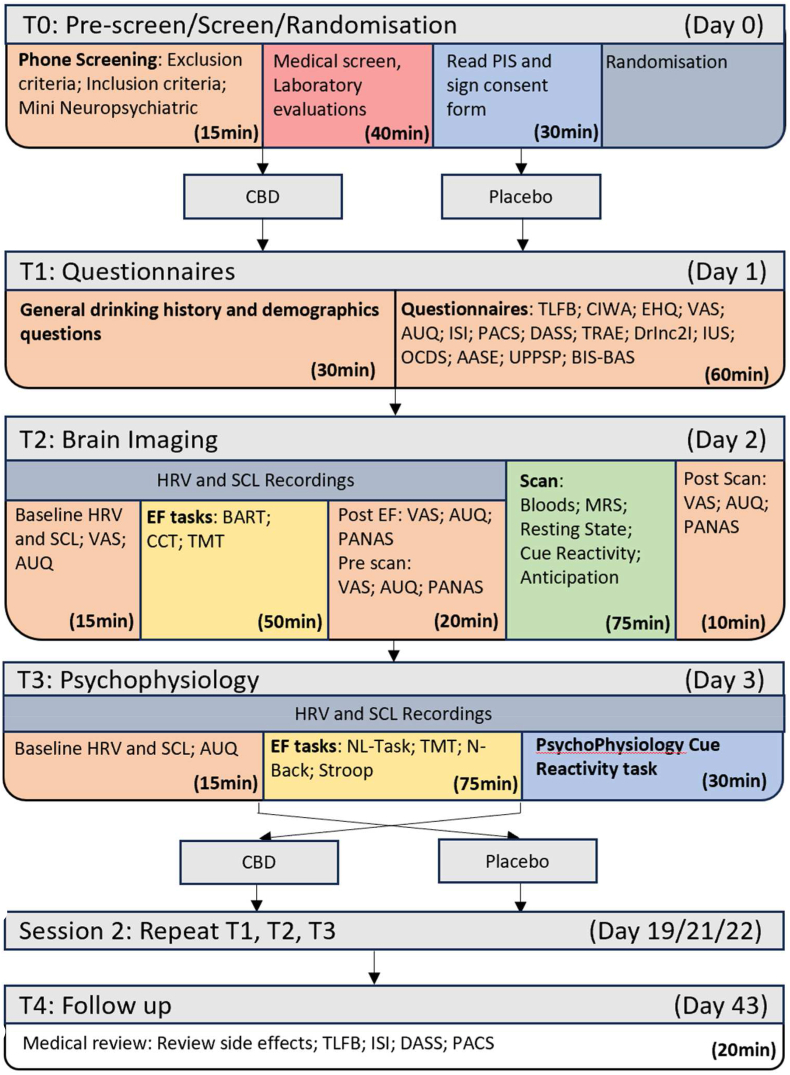
Study protocol flowchart: Timeline follow back (TLFB); Clinical Institute Withdrawal Assessment Alcohol Scale (CIWA); Edinburgh Handedness Inventory (EHQ); Visual analogue scale (VAS); Alcohol urge questionnaire (AUQ); Insomnia severity index ISI; Penn Alcohol Craving (PACS); Depression anxiety symptom scale (DASS); Tension Reduction Alcohol Expectancy Questionnaire (TRAE-Q); Drinker inventory of consequences (DrInc2I); Intolerance of uncertainty scale (IUS); obsessive compulsive drinking scale (OCDS); Alcohol Abstinence Self-efficacy scale (AASE); Urgency-Premeditation-Perseverance-Sensation Seeking-Positive Urgency (UPPS-P); Behavior Inhibition System and Behavior Approach System Scale (BIS-BAS); Heart Rate Variability (HRV); Skin conductance level (SCL); Ballon Analogue risk task (BART); Columbia card task(CCT); Trail making task (TMT); Positive and Negative Affect Schedule (PANAS); Magnetic Resonance spectroscopy (MRS); Number letter task (NL-Task).

### 2.2 Inclusion and exclusion criteria

*Inclusion criteria*: a) Male and female patients between the ages of 18 and 65 meeting DSM-V criteria for current alcohol use disorder; b) Adequate cognition and English language skills to give valid consent and complete research interviews; c) A blood alcohol concentration (BAC) reading of 0.00; d) Must have a stable residence and be able to identify an individual who could locate subject if needed; e) Women of child-bearing potential must be non-lactating, using birth control and have a negative pregnancy test; f) Willingness to give written informed consent.

*Exclusion criteria*: a) Active major psychological disorder associated with psychosis, significant suicide risk; b) Pregnancy or lactation - Women shall be advised to use reliable contraception for the duration of drug therapy and a urine pregnancy test will be performed where necessary; c) Dependence and/or regular use of any substance other than nicotine; d) Diagnosis of epilepsy, and/or current use of anti-epileptic drugs (AED); e) Liver failure with jaundice or prolonged INR above 1.3; f) Medical complications such as liver failure, cardiac ischemia or conduction abnormalities, renal impairment or unstable elevated vital signs (systolic blood pressure >180, diastolic blood pressure >120 or heart rate >150); g) Severe cognitive impairment or insufficient English or literacy to complete study processes; h) Concurrent use of drugs potentially exacerbated by CBD via CYP3A4, CYP2C9 and CYP 2C19 including cardiac medication (eg betablockers, calcium channel blockers and statins), macrolides and recent antihistamine use; i) **Claustrophobia; j) Extreme obesity; k) Previous brain surgery; l) Ever employed as a machinist, a welder or a metal worker; m) Metal items such as pacemakers; aneurysm clips in the brain; metal dental implants; metallic fragments in the eye or anywhere else; insulin pump; metal implants; hearing aid or a prosthetic device.**

### 2.3 Randomisation and allocation concealment

Participants will be randomised to receive 3 days of 800 mg CBD or matched placebo arms in a cross-over, double-blind, randomised trial. A random allocation procedure will be conducted by computer-generating a random table, using R (A programming language for statistical computing; RStudio Team (2020)). This randomisation table will be applied using REDCap (Research Electronic Data Capture; a secure web application for building and managing online surveys and databases) to randomly allocate participants to either treatment group. Pharmacists at the RPAH will be tasked with using REDCap's randomisation module to randomise participants to either active medication or placebo and subsequently dispense the medication. Prior to administration, participants will be required to detail any non-prescribed medication use or substance use each session before dispensing the medication by the research nurse. Researchers, clinicians, and participants will all be blinded to treatment allocation. In the event of a medical emergency that requires knowledge of the treatment condition in the opinion of the treating clinician at the time, the investigators will be able to contact the 24-h telephone service at the NHMRC Clinical Trials Centre to break the randomisation code for that individual.

### 2.4 Procedure

Participants will participate in a screening phase (over the phone and in-person medical screening), 6 days of in-person testing (T1-T3, repeated in a cross-over fashion), and one day of follow-up questions over the phone. T1 consists of a variety of questionnaires; T2 will include physiological recording during executive functioning tasks as well as a brain scan; T3 will consist of physiological recordings during executive functioning tasks and a cue-induced craving task. Specific measures are detailed in the measures section of this protocol paper. Fig. 2 demonstrates the overview of the procedure including when each task will be conducted. The order of days occurs from top to bottom (T0, T1, T2, T3, washout, crossover repeat, T4) while the order of procedures occurs from left to right. Additionally, a blood sample will be taken at the commencement of T1 and T2 followed by drug administration occurring at the commencement of each testing day (T1, T2, T3).

### 2.5 Pharmacotherapy schedule

*Cannabidiol:* Study medication will be administered onsite under observation in the form of four 200 mg, CBD oral soft gel capsules (manufactured by Linnea Natural Pharma Solutions) for a total dose of 800 mg per day, over three days. Medications will be delivered to the clinical trials pharmacy with accounting and reconciliation according to S4 principles according to established procedures. All dosage forms will be stored at 25 °C and in accordance with S4 poisons schedule guidelines. The pharmacist will be provided with access to the REDCap randomisation module and dispense the study medication without revealing the allocation to the participants or the study coordinator. The placebo capsules will be identical in appearance, taste, and composition except for the active ingredient of pure CBD. 800 mg capsules of CBD or placebo will be administered in the laboratory under supervision/observation once daily for 3 consecutive days starting on the first test session day, session 1 (T1). and patients will be monitored for side effects through each testing day.

This study will conduct the majority of testing under observation and participants will be monitored closely for adverse events during these testing days. Open ended questions regarding AEs will be assessed each day before dosing by the physician and nurse and systematic assessment for adverse events will be completed on the first day of the second arm (day 19) and at follow-up (day 43). If necessary, physician and/or psychiatric consultations will be arranged. Participants will be encouraged to call the investigator and/or the study physician if they experience any unusual or distressing symptoms during study involvement. Participants who experience mild or moderate side effects but wish to stay on the medication can have their dose reduced based on physician judgment. At the end of the study, participants will be asked to report any non-prescribed medication or substance use. In the event of a medical emergency where the participant needs to be unblinded, the lead investigator will be notified. Unblinding may occur by contacting the pharmacy to access the randomisation list. If a participant is unblinded, the details, date and reason for breaking the blind will be clearly documented.

### 2.6 Measures

*Phone Pre-Screening:* Before being invited to participate in the study, individuals who show interest in participating will be informed about the study in detail and initially screened for exclusion and inclusion criteria over the phone by the research team using a semi-structured interview. The study coordinator will then conduct further screening by administering the MINI Neuropsychiatric Interview [32].

*Medical/laboratory tests:* Participants will be screened for inclusion and exclusion criteria face to face and tested for objective measures of alcohol use. Firstly, participants will be assessed on medical history, including a history of any problems with alcohol withdrawal or risk factors for serious alcohol withdrawal such as unstable medical illness, concurrent medications, and psychiatric diagnoses. The physical examination at baseline will include blood pressure and cardiovascular observations, signs of alcohol-related liver disease, a brief neurological examination, and a mental state examination. Laboratory tests at baseline include urinalysis, urine toxicology, full blood count, liver function tests (LFTs: bilirubin, gGT, ALP, AST, ALT, albumin, protein), coagulation tests (INR, APTT), creatinine and phosphatidyl ethanol (PEth). Bicarbonate and electrolytes are checked to screen for metabolic acidosis. Blood samples will be collected and stored for biochemical, genetic, and molecular analysis in the current trial and for future use in ancillary studies.

*Blood sampling:* Blood sampling will be conducted at additional periods throughout testing for peripheral markers of inflammation, cortisol, and CBD plasma concentration.

*Questionnaires and assessment tools:* A set of general demographics and background drinking history questions (30 min) will be administered including demographics, medical history, personal and family history of AUD, and alcohol treatment history, as obtained in previous work [[33], [34], [35], [36]]*.* Additionally, Recent (last 28 days) alcohol consumption (frequency/quantity) assessed by the Timeline follow-back method (TLFB [37]; (ii) severity of alcohol dependence assessed by the Alcohol Dependence Scale (ADS; [38]); (iii) craving for alcohol measured by the Penn Alcohol Craving Scale (PACS [39]; (iv) the Drinker Inventory of Consequences (DrInC-L: baseline; R: follow-up [40]; provides a measure of the social, physical, and emotional consequences of alcohol use; (v) the Tension Reduction Alcohol Outcome Expectancies (TRAE) measures expectancies regarding the outcome of alcohol use [41]; (vi) the Depression Anxiety Symptom Scale (DASS) measures the severity of symptoms of depression, anxiety and stress [42]; (vii) sleep problems are assessed by the Insomnia Severity Index [43]; (viii) the Alcohol Abstinence Self-Efficacy (AASE) scale measures perceived self-efficacy [44]; (ix) Obsessive Compulsive Drinking Scale [45] measuring drinking obsessionally and compulsivity; (x) the behavioural approach and avoidance (BIS/BAS [46]; (xi) the Intolerance of Uncertainty Scale (IUS [47]; measuring reactions to ambiguous situations (xii) the Urgency Premeditation Perseverance Sensation-Impulsivity Scale [48] measuring impulsivity; (xiii) expectancy of alcohol effects and urges to drink (AUQ [49]; (xiv) the Positive and Negative Affect Schedule measuring positive and negative mood states (PANAS; [50]); (xv) Visual Analogue Scales (VAS) assessing alcohol craving, thirst, and anxiety. Additionally, Participants will also be required to answer 10 questions from the Leeds Sleep Evaluation Questionnaire (LSEQ [51]; and 17 questions from the Consensus Sleep Diary (CSD-M [52]; administered through the SEMA3 application [53].

#### 2.6.1 Cognitive and executive function

A suite of cognitive tasks will be used to assess cognitive capacity including the: (i) Number–letter task (adapted from Ref. [54]) to assess working memory; (ii) Stroop task [55] to assess inhibitory control; (iii) N-back task [56,57] to assess working memory capacity; (iv) Balloon Analogue Risk Task (BART,[58]). to assess self regulation and adaptive risk-taking; (v) Columbia Card Task, Hot Version (CCT; [59]) assessing inhibition, working memory updating, task-set switching, and attention; (vi) Trail making test (TMT: Part A and B [60]) assessing set-shifting flexibility, attention, and inhibition.

#### 2.6.2 Imaging

MRI data will be acquired on a 3 T GE Discovery 750 scanner (GE Healthcare, Milwaukee, Wisconsin, USA), using a 32-channel phased array head coil. The imaging order will be as follows i) 267 s of T1-weighted (T1-w) structural scan, ii) 544 s of MRS to determine levels of GABA+, glutathione, and ethanol, iii) 136 s B_0_ Map, iv) 620 s multi-echo (ME) resting state), v) 645 s ME fMRI alcohol cue reactivity task, vi) 633 s single-echo fMRI anticipation using anxiety inducing/anticipatory and matched neutral cues stimuli.

*Structural:* A T1-weighted (1-mm^3^ voxel resolution) structural scan will be acquired for each subject for voxel placement co-registration (TR, 7200 ms; TE, 2.7 ms; 176 sagittal slices; 1 mm thick; no gap; 256 × 256 × 256 matrix).

*Proton Magnetic Resonance Spectroscopy (*^*1*^*H-MRS):* An edited ^1^H-MRS using Hadamard Encoding and Reconstruction of Mega-Edited Spectroscopy (HERMES; [61]) sequence will be acquired. This acquisition is optimized to simultaneously edit for gamma-aminobutyric acid, glutathione, and ethanol quantification [62,63]. The voxel will be placed in the dorsal ACC with the long edge of the voxel along the genu of the corpus callosum (voxel size 4 × 2 × 3 cm^3^). The acquisition parameters for the HERMES are TR/TE = 2000/80 ms, 256 VAPOR water-suppressed scan averages, 16 unsuppressed scan averages, number of data points = 4096, spectral width = 5000 Hz, acquisition time 544 s. The dorsal ACC is chosen given high relevance in the reward/motivation circuit [64].

*Resting-state fMRI (rs-FMRI) sc*an: BOLD signal will be recorded using a Multiecho echoplanar imaging (ME EPI) sequence which enables improved signal to inferior regions (e.g., VMPFC etc) which generally suffer from signal loss [65]. ME EPI allows for the BOLD signal to be recovered at multiple echo times per radio frequency excitation pulse. Hyperband Multi-echo EPI (HyperMEPI; GE Healthcare, Waukesha, WI, USA) will be used to capture multiphase volumes of whole brain, comprising 45 axial slices collected in an ascending interleaved fashion angled 15° from the AC-PC line superior to inferior using a Gradient Echo pulse sequence with 3 echoes (TR: 2000 ms, TE_1_/TE_2_/TE_3_ = 0.01/0.025/0.04 s, Flip Angle:70°, FOV:220 mm, matrix of 64 × 64, slice thickness 3 mm; slice gap 0.4 mm; with a voxel resolution 3.44 × 3.44 × 3.44 mm3). The B_0_ map will be acquired using the same parameters as above except the following: TR = 1000 ms, TE = 4.6 ms, flip angle 30°, FOV: 220 mm, matrix 64 × 64, and bandwidth 62.5.

#### 2.6.3 Functional magnetic resonance imaging (fMRI)

*Cue Reactivity Task:* Participants will complete a visual cue reactivity task adapted from Ref. [66] to measure alcohol cue-elicited brain activity. Stimuli comprise two types: alcohol-related pictures depicting types of alcohol (larger/wine/spirits) and drinking situations; and a control type comprising validated neutral pictures matched for colour and complexity. Images will be presented for 6.6 s in blocks of 3 images of the same type. 10 alcohol stimuli and 6 neutral blocks will be presented throughout the experiment with stimuli and block order will be randomised (16 total blocks, 645 s). Following each block, an 11-point visual analogue craving scale is presented and participants respond using an MRI-compatible two-button response pad (Cedrus Corporation; San Pedro) within a 10 s window.

*Fear Anticipation Continuous Performance Task (FCPT):* A novel fear response task consisting of a continuous performance task (CPT) with an intermittent presentation of high-threat and low-threat cues will be used to evaluate brain activity elicited by anticipation to fear-inducing cues. This task utilises stimuli from the Nencki Affective Picture System (NAPS [67]; which is a validated set of images that reliably elicits a fear response. Specific images that will be selected for high and low-threat blocks are listed in Table 2 in the appendix. Participants also complete the CPT to maintain attention, indicating the direction of 3–6 arrows pointing left or right presented on a grey background, along with a 440 Hz tone, using the response pad buttons (arrow block). These arrow blocks are followed by high-threat or low-threat blocks, in which participants are presented with 3 arrows along with either high-threat (‘threat’ 1000 Hz + Orange background) or a low-threat (‘safe’ 250 Hz + Blue background) signal stimulus followed by presentation of a fearful or neutral pictorial unconditioned stimulus (US) respectively. The US images comprise 18 fear cues (of animals, objects, humans, and faces) and 18 control images matched for valence and arousal dimensions. The number of arrows is randomised per arrow block, with ≤2 same blocks presented consecutively, and US block type order (either fearful or neutral) with ≤3 same US type presented consecutively, and US block order randomised per participant.

*Psychophysiological alcohol cue reactivity*: The psychophysiological cue reactivity (CR) task has been described previously [68]. The CR contains 5 consecutive stages including baseline, juice cue, recovery 1, alcohol cue, and recovery 2; (5 min stages, total = 25 min). In cue reactivity stages (cue exposure stages) either a bottle of a novel, non-sweet juice (carrot, control) or a bottle containing the participants preferred alcoholic drink will be placed in front of participants with relevant juice/beer/wine glasses. Alcohol cues will either be red/white wine or lager depending on which of these the participant most commonly consumes. We were not permitted to provide other types of alcohol which could potentially be a limitation however our initial sampling of the target population revealed that beer and wine were the types most commonly consumed. Vignettes that provide context to enhance drink cue craving will be presented before the participants are instructed to pour, hold, and smell the beverage for 5 min. During this alcohol cue presentation, a previously hidden simulated bar will be revealed to enhance external contextual cue craving. During the 5-min baseline and recovery periods, participants will view neutral landscape videos set to classical music.

Throughout the psychophysiological CR paradigm, MLT117F GSR Electrodes (ADInstruments; Bella Vista, Australia) will be placed on the middle phalanges on the II and III fingers of the participant's non-dominant hand. A FE116 GSR Amplifier (ADInstruments; Bella Vista, NSW, Australia) will then be used to amplify the skin conductance signal. Additionally, electrocardiogram (ECG) data will be recorded using a three-lead ECG with Ag/AgCl electrodes. These electrodes will be placed on the non-dominant wrist (as a ground electrode) and two above the cubital fossa on each arm. This ECG signal will then be amplified using an ML408 Dual Bioamp/Stimulator (ADInstruments; Bella Vista, NSW, Australia). Both amplifiers will be connected to a PC operating LabChart Pro 7.3.7 software (ADInstruments, 2012) via a PowerLab 8/25 System (ADInstruments; Sydney, Australia) and sampling at a rate of 1000 Hz/s. Additionally, in-between cue exposure phases; AUQ, VAS and PANAS will be used to record craving as a self-measure and applied in data analysis.

*Brain metabolites (MRS):* The open-source *“*Osprey” toolbox [69] will be used in MATLAB to process and quantify the presence of metabolites (in ppm) for the HERMES acquisition. Metabolites will be corrected for tissue relaxation factors [70,71], and in the case of GABA, the alpha correction will be applied [72]. Signal-to-noise and fit metrics, along with visual inspection, will be used to ensure quality of the data. Generalised estimation equations (GEEs) will be implemented using the CrossCarry package [73] using R software (Version 4.0.3), which allow for the estimation of both simple and complex carryover effects within the context of a crossover design with repeated measures. GEE models are advantageous compared to parametric approaches as they account for the order and carryover effects with effects averaged across groups [73,74]. Session order and carryover effects will be explicitly modelled using Crosscarrys createCarry. Additionally, covariates reflecting previous day drinking (PDD) and percentage of heavy drinking day during the previous two weeks will be modelled given the effects of recent drinking on neurometabolite concentration [75].

#### 2.6.4 fMRI

*Preprocessing:* fMRIPrep [76,77], will be used for both anatomical and structural image preprocessing. Intensity non-uniformity correction will be applied to T1-w images which will then be skull-stripped. After these T1-w images are used in brain surface reconstruction, volume-based structural images will be segmented and normalised into MNI space. fMRIPrep will additionally be used for cue reactivity and resting state ME EPI preprocessing. Images acquired during the ME EPI sequences will be distortion-corrected using the B0 field maps, motion-corrected, co-registered to the T1-weighted structural data, normalised to MNI space, and projected to cortical surface. Functional time series will then be resampled to FreeSurfer's (FreeSurfer 6.0.1, surfer.nmr.mgh.harvard.edu) fsaverage space. Further, distortion correction, motion correction, slice timing, co-registration, spatial normalisation, and smoothing will be applied for FCPT single-echo preprocessing. Task-based volumes will be excluded if they demonstrate framewise displacement of greater than 4 mm.

*Post Processing:* Cue reactivity and FCPT data will be post-processed separately using SPM. To improve sensitivity during group analysis, functionally resampled images will be smoothed with a full-width half maximum (FWHM) 8 mm Gaussian kernel. eXtensible Connectivity Pipelines (XCP-D) [78,79] will be used to further process resting state data. Resting-state volumes will be excluded if they demonstrate framewise displacement of greater than 4 mm. Additionally, nuisance regressors based on the ‘36P’ strategy [78,79] will be regressed out. Using the XCP-D output from each resting state acquisition a Fisher's r-to-z transformation will be applied to Pearson correlation coefficients for each resultant ROI-to-ROI connectivity matrix cell using the Schaefer 17-network 400 parcel atlas. This will provide us with 44 (22 participants with two sessions) symmetrical functional connectivity matrices with 400 × 400.

#### 2.6.5 fMRI statistical analysis

Data analysis will be conducted using SPM12 with two levels. Within the first level (session-specific) analysis two conditions will be modelled for each task: alcohol for cue reactivity or high threat anticipatory cues for FCPT; and control cues (low threat and cue reactivity control cues; matched cues for novelty and visual complexity of the stimuli). These conditions will be modelled as a box-car function convolved with the canonical haemodynamic response. Additionally, for both tasks six motion correction parameters, VAS blocks, and CPT arrow (during FCPT analysis) blocks will be included as regressors of no interest, and fixation crosses will be considered an implicit baseline.

For our second-level random-effects analysis we will be using the MarsBar toolbox ([80]) and extract unweighted beta estimates (*β*) within ROIs specific to each task. A priori regions of interest (ROIs) will be used, including the dorsolateral PFC, left and right caudate, and bilateral ventromedial PFC given these regions are shown to be relevant in alcohol cue studies [81,82]. For the FCPT ROIs will include the amygdala, parahippocampal gyrus, insula, ventral striatum, and hypothalamus given their relevance to fear anticipation [83]. Regions will be defined using the Brainmap database [84] and relevant *β* will be collected for both sessions of each participant, and drug differences will be analysed using GEEs using the CrossCarry package in R software (Version 4.0.3). GEEs will be modelled for alcohol and control conditions for cue reactivity, and high and low threat cues for anticipation task will be across both sessions. Individual contrasts within condition will be selected as comparisons between conditions (eg Alcohol against control) have been shown to have low reliability in repeated measures designs [85]. Session order and carryover effects will be explicitly modelled using Crosscarrys createCarry, and PDD will be modelled as a covariate given its effects on functional brain activity [86]. We will then use the Simple Interactive Statistical Analysis Bonferroni tool (http://www.quantitativeskills.com/sisa/calculations/bonfer.htm) to account for correlation between ROIs. For task-based results we will report beta mean correlation coefficients, and resulting in an equivalent corrected alphas with a threshold of P < 0.05.

#### 2.6.6 Resting-state data analysis

Using the CONN toolbox, functional connectivity will be extracted from the resting state data [87] version 22a (Nieto-Castanon & Whitfield-Gabrieli, 2021). To compare CBD and Placebo (CBD > placebo [i.e., 1 -1]) functional connectivity we will use GLM*,* 44 (22 participants with two sessions) symmetrical functional connectivity matrices. Additionally, parametric multivariate statistics will then be applied to identify individual ROI contributions. Further, a FDR-corrected ROI-level p-value (P_FDR_ <0.05, p < 0.1) MVPA omnibus test [88] will be used to examine individual ROI-ROI connections.

*Psychophysiology*: GEEs will be used to assess session differences and treatment effects for continuous variables of psychophysiological indices HF-HRV and SCL, and AUQ craving scores, with planned contrasts comparing specific key stages of the cue reactivity task (i.e., baseline versus cue presentations, neutral and alcohol cues, cue presentations versus recovery periods, alcohol cue presentation versus alcohol recovery period). Session order and carryover effects will be explicitly modelled using Crosscarrys createCarry, andPDD will be modelled as a covariate given its effects on skin conductance [89], cardiovascular responses [90,91], and craving and mood measures [92].

*Follow-up data:* Outcomes variables (alcohol consumption, craving, mood, sleep) will be summarized by calculating means, standard deviations, and percentile ranges for all continuous variables and by calculating proportions for all categorical variables. A one-way analysis of variance (ANOVA) will be used to assess group differences for continuous variables. Nominal variables will be analysed via chi-square tests of independence. Mixed models will be conducted to examine differences from baseline (CBD vs PL as main group effects).

*Sample Size:* The sample size of N = 22 with 2 observations per participant is calculated based previous literature implementing a similar design and statistical analysis [93]. Given the complexity of conducting an a priori power analysis we used G*power to conduct a power analysis using an analytic approach with less statistical power (repeated-meausre ANOVA). The analysis indicated that a total sample size of 22 participants was required to achieve a power of 0.85. The critical F-value for this analysis was 4.32479. These results ensure that the study has an adequate sample size to detect a medium effect size with a high probability of success (power = 0.85) while maintaining an acceptable Type I error rate (α = 0.05).

## 3 Discussion

This study intends to examine early signs of efficacy of CBD as a pharmacotherapy in the management of AUD by identifying modulation in relevant neurobiological and psychophysiological systems.

The primary interest of the research is mechanistic and therefore MRI paradigms investigating brain metabolite levels (MRS), functional brain connectivity through (rsfMRI) and functional activity in areas associated with AUD-related characteristics will be applied. We will also use heart rate variability and skin conductance paradigms to investigate parasympathetic and sympathetic responses to alcohol cue presentations. Secondary outcomes, considering the changes to clinical measures associated with AUD (including alcohol consumption, craving, mood, and sleep), will additionally be measured,

CBD could reduce alcohol craving and seeking due to moderating responses to alcohol and stress cues, normalising dysregulated neurobiological systems and/or improving relevant clinical characteristics that lead to relapse such as sleep and mood disturbances. Compared to other medications used for the management of addiction, CBD has been demonstrated to be particularly safe with less severe side effects and few contraindications [94] which may lead to better treatment adherence [95]. CBD may also offer potential protection from alcohol-related liver and brain damage due to anti-inflammatory and antioxidant properties. By improving our understanding of CBD in relation to AUD-related neurobiological, cognitive, and clinical characteristics, this study will provide key information with regards to the potential of CBD as a pharmacotherapy for the management of AUD.

## Trial status

The trial is in the recruitment phase.

## Ethics approval and consent to participate

Ethics approval for the study has been granted by The 10.13039/501100024053Sydney Local Health District Ethics Review Committee (X19-0416 & HREC/16/RPAH/283). Participants are provided with an approved plain language information sheet and provide written consent to participate. The protocol is version 6 (X19-0416, 27/7/2022). The trial is sponsored by Sydney Local Health District and was registered with the NIH clinical trials registry retrospectively on May 24, 2022: NCT05387148 (https://classic.clinicaltrials.gov/ct2/show/NCT05387148). Participants are advised of their right to seek compensation from the study sponsor in the event of harm arising from trial participation. Protocol amendments to the clinical protocol will be updated to the clinical trials registry following approval by the corresponding ethics review committee.

## Consent for publication

There are no restrictions on publication from the sponsor or other parties. Publications will be authored by the investigators and authorship offered according to NHMRC criteria.

## Availability of data and materials

De-identified data will be published on the www.clinicaltrials.gov website.

## Funding

This project is funded by a 10.13039/501100000925National Health and Medical Research Council grant APP2009851 (PH, KM) and an10.13039/100015539Australian Government Research Training Program (TH). The funders do not have any role with regards to study design, implementation of the project, data analysis or the decision to submit this report for publication.

## Trial registration

NCT05387148.

## CRediT authorship contribution statement

**Tristan P. Hurzeler:** Writing – original draft, Resources, Project administration, Methodology, Investigation, Conceptualization. **Warren Logge:** Writing – review & editing, Supervision, Methodology, Formal analysis, Conceptualization. **Joshua Watt:** Writing – review & editing, Resources, Investigation. **Marilena M. DeMayo:** Writing – review & editing, Supervision, Software, Methodology, Formal analysis. **Anastasia Suraev:** Writing – review & editing, Resources, Conceptualization. **Iain S. McGregor:** Writing – review & editing, Resources, Conceptualization. **Paul S. Haber:** Writing – review & editing, Supervision, Resources, Investigation, Funding acquisition, Conceptualization. **Kirsten C. Morley:** Writing – review & editing, Supervision, Methodology, Investigation, Funding acquisition, Conceptualization.

## Declaration of competing interest

The authors declare that they have no known competing financial interests or personal relationships that could have appeared to influence the work reported in this paper.

## Figures and Tables

**Fig. 3.1:**
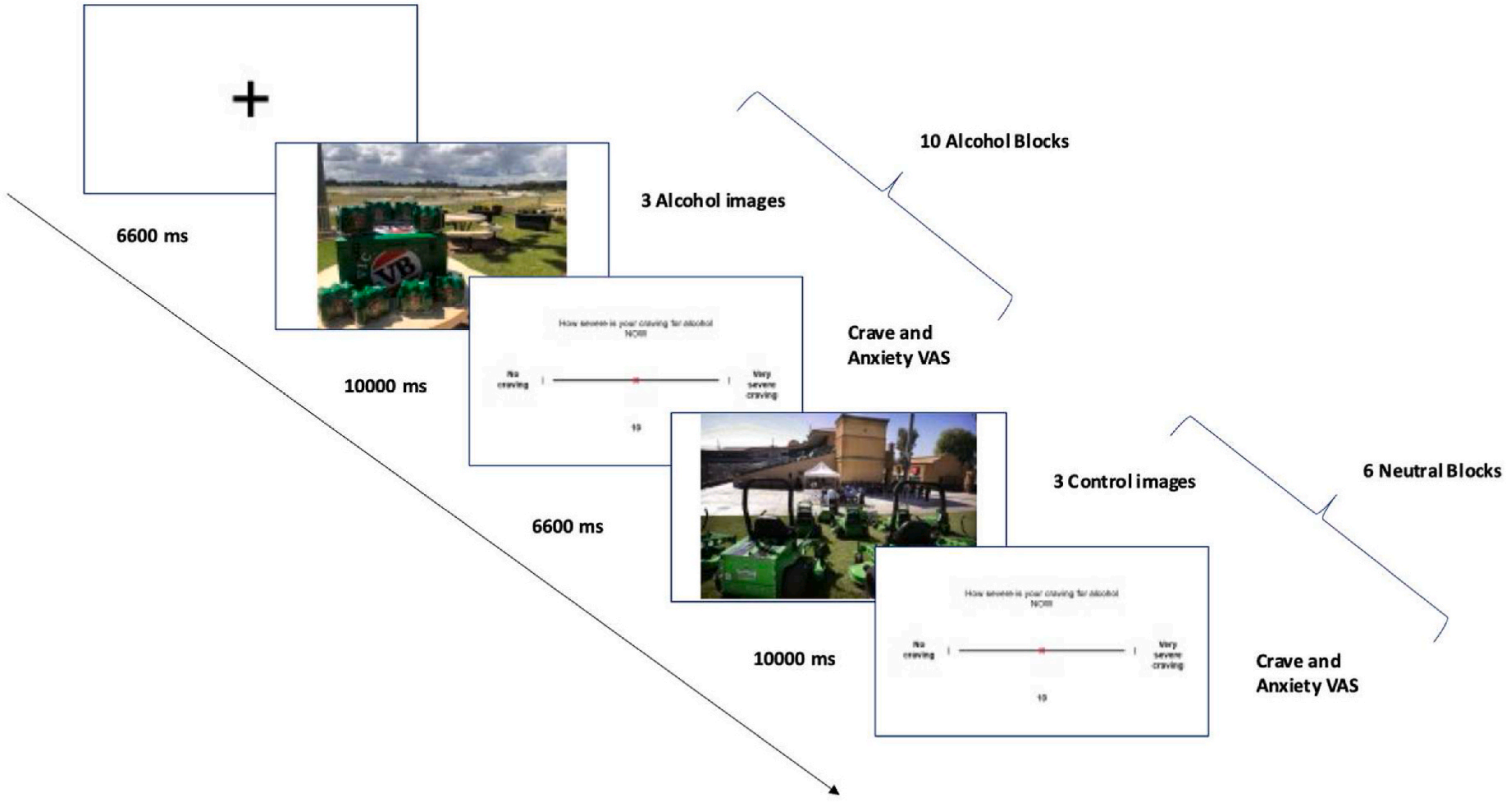
fMRI Cue Reactivity Task.

**Fig. 3.2:**
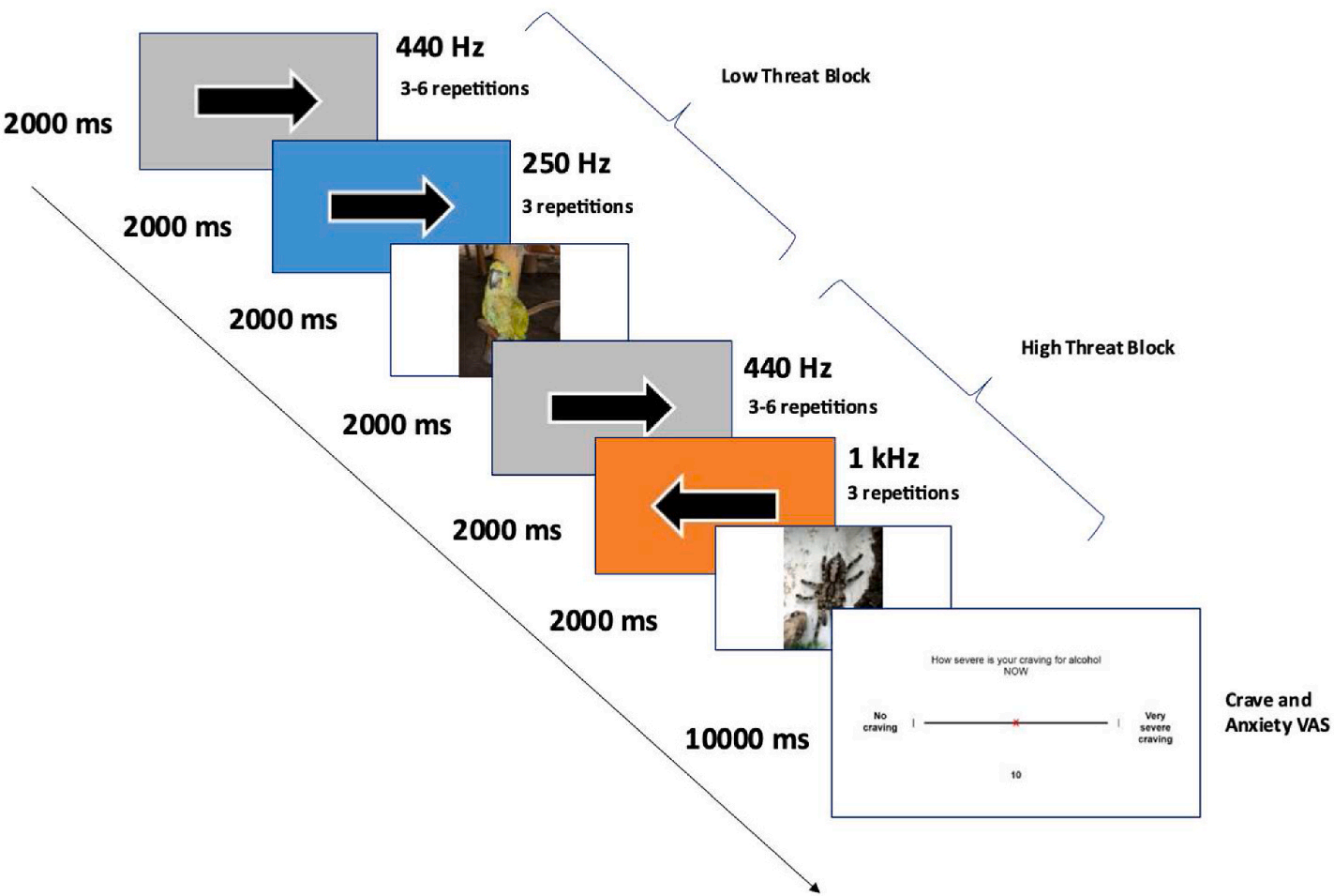
FCPT.

**Table 2:** Nencki Affective Picture System (NAPS) images selected per block. The items position in high threat and low threat list are matched on valence and arousal (004 matches 178; 006 matches 179 ect)

Block	NAPS image number
High Threat	004, 006, 007, 011, 022, 030, 052, 055, 058, 073, 076, 144, 009, 003, 283, 285, 038, 074
Low Threat	178, 179, 165, 122, 213, 197, 215, 117, 212, 195, 173, 118, 321, 069, 078, 264, 183, 180

## Data Availability

No data was used for the research described in the article.
